# *UCN*-Centric Prognostic Model for Predicting Overall Survival and Immune Response in Colorectal Cancer

**DOI:** 10.3390/genes15091139

**Published:** 2024-08-29

**Authors:** Jia Liu, Feiliang Zhong, Yue Chen

**Affiliations:** 1 Key Laboratory of Molecular Microbiology and Technology of the Ministry of Education, Department of Microbiology, Frontiers Science Center for Cell Responses, College of Life Science, Nankai University, Tianjin 300071, China; liujialeo@mail.nankai.edu.cn; 2 College of Biotechnology, Tianjin University of Science and Technology, Tianjin 300457, China

**Keywords:** colorectal cancer, prognostic model, TCGA, *UCN*, single-cell transcriptome, immunity

## Abstract

Colorectal cancer (CRC), a prevalent malignancy, ranks third in global incidence and second in mortality rates. Despite advances in screening methods such as colonoscopy, the accurate diagnosis of CRC remains challenging due to the absence of reliable biomarkers. This study aimed to develop a robust prognostic model for precise CRC outcome prediction. Employing weighted co-expression network analysis (WGCNA) and Cox regression analysis on data from The Cancer Genome Atlas (TCGA), we identified a panel of 12 genes strongly associated with patient survival. This gene panel facilitated accurate CRC outcome predictions, which is also validated via the external validation cohort GSE17536. We conducted further investigations into the key gene, *urocortin* (*UCN*), using single-cell transcriptomic data and immune infiltration analysis in CRC patients. Our results revealed a significant correlation between high *UCN* expression and the reduced prevalence of key immune cells, including B cells, CD4+ cytotoxic T cells, CD8+ T cells, and NKT cells. Functional experiments showed that *UCN* gene interference in the CRC cell lines significantly decreased cancer cell proliferation, underscoring *UCN*’s role in intestinal immunity modulation. The *UCN*-centric prognostic model developed enhances prognosis prediction accuracy and offers critical insights for CRC diagnosis and therapeutic interventions.

## 1. Introduction

Colorectal cancer (CRC) represents a significant global health concern, ranking as the third most common malignancy and the second leading cause of cancer-related deaths worldwide [1]. Annually, CRC accounts for over 1–2 million new cases and approximately 600,000 deaths [2]. The development of colonic tumors typically progresses through a well-documented normal–adenoma–carcinoma sequence, involving a series of histological, morphological, and genetic changes over time [3]. Early and precise screening and diagnosis are critical in reducing the incidence and progression of CRC. Despite this, traditional diagnostic methods, such as colonoscopy—the gold standard—face limitations due to their invasiveness [4], the differences in doctor’s subjective judgment, and the difficulty in detecting precancerous stages before extensive malignancy develops. These challenges highlight the critical need for reliable diagnostic markers that can accurately predict disease progression in CRC patients.

Next-generation sequencing technologies, especially RNA sequencing (RNA-seq), have advanced our understanding of gene expression dynamics throughout CRC progression [5]. Integrating clinical data with the extensive RNA-seq datasets from CRC patients facilitates the development of prognostic models crucial for disease prevention, diagnosis, and therapeutic strategies [6]. This approach can identify molecular markers capable of accurately forecasting disease trajectory and prognosis, providing essential support for clinical decision-making [7], compensating for the limitations of human judgement in colonoscopy and helping to find accurate targets for drug development. For example, immune gene signatures and fatty acid metabolism-related gene signatures are used to predict CRC patient prognosis and as an indicator to evaluate immunotherapy response [8,9]. Prognostic model construction methods such as LASSO Cox regression and nomograms are effective, whereas they may have limitations in predictive accuracy when used independently [10]. Combining these diverse modeling techniques can enhance the precision of prognostic models, thereby offering profound insights into disease management and the customization of therapeutic interventions.

In this study, we aimed to identify a disease progression gene signature for the prediction of CRC, combining LASSO Cox regression and nomograms prognostic models, and furthermore validate the functions of the key gene in this signature in the immune microenvironment of CRC. A panel of twelve genes was identified from the TCGA-COAD database through weighted gene co-expression network analysis (WGCNA) and LASSO Cox regression. This model, coupled with a corresponding nomogram, showed exceptional capability in predicting CRC patient outcomes. Our exploration into immune infiltration and the roles of the key gene, *UCN*, in the tumor microenvironment revealed the following notable finding: Increased *UCN* expression correlates with reduced immune cell presence in CRC patients, as indicated by single-cell RNA sequencing data. Specifically, targeting the *UCN* gene significantly reduced proliferation in colonic cancer cell lines, underscoring its critical function in modulating the immune landscape of intestinal tumors. Thus, the *UCN*-centric prognostic model stands out as a promising tool, enhancing the accuracy of prognostic predictions for CRC patients while illuminating new diagnostic and therapeutic possibilities. These insights establish *UCN* as a potential novel biomarker and a key player in regulating immune responses in CRC, opening avenues for targeted therapy development.

## 2. Materials and Methods

### 2.1. Cell Lines

The colon cancer cell lines Caco-2 and HT-29 were procured from the American Type Culture Collection (ATCC, Manassas, VA, USA). These cells were cultured in Minimum Essential Medium (MEM, Gibco, Thomastown, VIC, Australia) and McCoy’s 5A medium (Gibco), each supplemented with 10% fetal bovine serum (FBS, Gibco) and 10 U/mL Penicillin-Streptomycin (Gibco). Cultivation was performed in a CO_2_ incubator at 37 °C with a 5% CO_2_ atmosphere.

### 2.2. Data Source

The bulk RNA-seq dataset, TCGA-COAD, encompassing 41 normal samples and 471 colorectal cancer samples, was obtained from The Cancer Genome Atlas (https://portal.gdc.cancer.gov/, accessed on 1 January 2023). This TCGA-COAD served as a training cohort, and 200 samples were randomly selected from TCGA-COAD as an internal validation cohort. GSE17536, including 177 colorectal cancer samples, was accessed from the Gene Expression Omnibus (GEO) database (https://www.ncbi.nlm.nih.gov/geo, accessed on 15 January 2023) and served as an external validation cohort. Additionally, the CRC single-cell RNA-seq (scRNA-seq) dataset GSE161277 was also accessed from the GEO database (https://www.ncbi.nlm.nih.gov/geo, accessed on 15 January 2023). This dataset included 3 normal colonic tissue samples (number of cells was 14,170), 3 adenoma tissue samples (number of cells was 15,465), and 3 carcinoma tissue samples (number of cells was 14,530).

### 2.3. Weighted Gene Co-Expression Network Analysis (WGCNA)

Utilizing the TCGA-COAD cohort, differentially expressed genes (DEGs) were identified (adjusted *p*-value < 0.01, |log_2_FoldChange| > 2) via the DESeq2 package in R software. WGCNA was performed to discern modules of correlated genes and to pinpoint survival-related biomarkers [11,12]. The gene expression matrices were converted into matrices containing the similarity of the paired Correlation Coefficient test results. Subsequently, using the represented scale-free gene co-expression topological approach to investigate the smallest possible value ensured that the adjacency matrix met the scale-free topology criteria. Following this, Topological Overlap Matrix (TOM) and dissimilarity TOM (dissTOM) were established using TOM similarity and dissimilarity modules, respectively, for the next step. Ultimately, the module identification was performed using dynamic tree cut, setting the minimum module size to 30. Modules exhibiting high similarity scores were amalgamated, applying a threshold value for each dataset.

### 2.4. Least Absolute Shrinkage and Selection Operator (LASSO)

In constructing the prognostic model, genes significantly correlated with survival outcomes were first identified using a univariate Cox regression model. Thereafter, reliable predictors were selected through Least Absolute Shrinkage and Selection Operator (LASSO) analysis. Each patient’s risk score in the TCGA database was calculated using the formula risk score = Σ coefficient_mRNAn_ * expression level_mRNAn_. Subsequent analysis further examined the correlation between the risk score and the prognosis of the patients.

### 2.5. Nomogram Construction and Conformation

Cox proportional hazard regression models were used to carry out univariate and multivariate analyses, with the goal of determining the hazard ratio and the corresponding 95% confidence interval (CI) for all potential risk factors. The forward stepwise selection method was then applied to identify all independent risk factors via the multivariate Cox proportional hazard models. A nomogram was constructed by integrating all independent risk factors to predict 1-year, 3-year, and 5-year overall survival (OS) and cancer-specific survival (CSS). This was performed using the RMS R package (https://cran.r-project.org/web/packages/rms/, accessed on 10 February 2023). The nomogram’s discrimination was evaluated using Harrell’s concordance index (C-index). Calibration curves were used to gauge the consistency between the actual prognosis and the nomogram-predicted survival probability of the model.

### 2.6. ScRNA-Seq Data Analysis

The Seurat R package (version 4.3.0) offers an extensive pipeline for scRNA-seq data analysis [13]. Data preprocessing involved transforming raw scRNA-seq data into gene expression matrices, eliminating lowly expressed genes, and correcting for batch effects. Quality control measures entailed assessing the expression levels of mitochondrial genes and the number of expressed genes, with the subsequent removal of low-quality cells. The retained cells were then clustered using the K-means algorithm, and the optimal number of clusters was ascertained via the Elbow method. Cluster labels were assigned based on the most prominently expressed marker genes for each cluster. Differential gene expression analysis was conducted between varying cell types or states using the Wilcoxon rank-sum test. Genes exhibiting an adjusted *p*-value of less than 0.05 and a fold change of greater than 1.5 were considered significantly differentially expressed. Cell types were subsequently annotated by comparing the gene expression profiles of each cell with known cell type markers. The cell type annotations were verified by comparing these results with published scRNA-seq datasets. Overall, the Seurat R package delivers a comprehensive pipeline for scRNA-seq data analysis, which can be utilized across various scRNA-seq datasets to gain insights into cellular heterogeneity and gene expression patterns in complex biological systems.

### 2.7. Immune Cell Infiltration Analysis

The immune score for each sample was determined using the ESTIMATE package [14]. The relative abundance of various cell types within each colon tumor tissue, given different *UCN* and *GABRD* mRNA expression statuses, was computed using CIBERSORT1. The relative proportion of diverse tumor-infiltrating lymphocytes (TILs) was inferred for each cancer type using Gene Set Variation Analysis (GSVA) based on the immune-related gene expression profile of 14 cell types derived from the single-cell RNA sequencing of colon cancer tissues in this study. The correlation between cell populations and *UCN* or *GABRD* expression was assessed using Spearman’s test. Further, the differential expression of *UCN* or *GABRD* between the tumor and adjacent normal tissues across all the TCGA tumors was investigated utilizing the Tumor Immune Estimation Resource (TIMER) in this study.

### 2.8. Western Blotting

The extraction of total proteins from cells was performed using the RIPA lysis buffer (Solarbio, Beijing, China) supplemented with 1 mM PMSF (Solarbio) and a protease inhibitor cocktail (Solarbio). The protein concentration was determined using the bicinchoninic acid (BCA) Kit (Thermo Fisher Scientific, Waltham, MA, USA). Samples were then heated at 100 °C for 10 min, separated by 10% SDS-PAGE electrophoresis, and transferred to polyvinylidene difluoride membranes. The membranes were blocked with 5% bovine serum albumin (BSA), incubated with primary antibodies overnight at 4 °C, and then secondary antibodies for 1 h at room temperature. The protein signals were visualized using an ECL kit.

### 2.9. RNA Isolation and Quantitative Real-Time PCR

Total RNA was extracted from cells using the Trizol Reagent (Thermo Fisher Scientific), following the manufacturer’s guidelines. Complementary DNA (cDNA) was subsequently synthesized using the cDNA Synthesis SuperMix Kit (TransGen, Beijing, China), again adhering to the manufacturer’s instructions. Quantitative PCR (qPCR) was performed utilizing the PerfectStart Green qPCR SuperMix Kit (TransGen) and the CFX96 Connect System (Bio-Rad, Lunteren, The Netherlands). Relative mRNA expression levels were determined using the ΔΔCt method, and normalized to the expression of the housekeeping gene, *GAPDH* [15]. The sequences for the primers used are listed in Supplementary Table S1.

### 2.10. Cell Viability Assay

Caco-2 and HT-29 cells were transfected with *UCN*/*GABRD* shRNA for 48 h in 96-well plates. Following this, 10 μL of the CCK8 reagent was added to the culture media and the plates were incubated for an additional 4 h. Cell viability was then assessed by measuring absorbance at a wavelength of 450 nm, in accordance with the manufacturer’s instructions.

### 2.11. Statistical Analysis

Unless otherwise stated, all values are presented as means ± SEM. To evaluate significant differences between two groups, two-tailed Student’s *t*-tests were employed, while significant differences among multiple groups were assessed via one-way analysis of variance (ANOVA). All statistical analyses were conducted using GraphPad Prism (version 8.0.2). *p* < 0.05 was considered statistically significant.

## 3. Results

### 3.1. Identification of Survive-Related Gene Modules in CRC Using WGCNA

The RNA-seq expression dataset from the TCGA-COAD, encompassing 19,568 mRNA transcripts, was analyzed for 471 colorectal adenocarcinoma (COAD) patients and 41 healthy subjects (Figure S1A). Following data normalization, the DESeq2 R package was utilized for differential expression analysis, setting thresholds at |log_2_FoldChange| > 2 and an adjusted *p*-value < 0.01. This analysis identified 2057 genes with significant expression differences between tumor and normal samples, including 1066 upregulated and 991 downregulated genes (Figure S1B). To explore gene expression patterns related to clinical aspects of CRC, we applied the Weighted Gene Co-Expression Network Analysis (WGCNA). This included constructing a co-expression network from 512 colonic tissue samples in the TCGA-COAD dataset. Hierarchical clustering analysis confirmed the absence of conspicuous outlier samples (Figure S2A). Pearson’s correlation coefficient was used to compute inter-gene distances, leading to the establishment of a scale-free network (Figure S2B).

**Figure 1:**
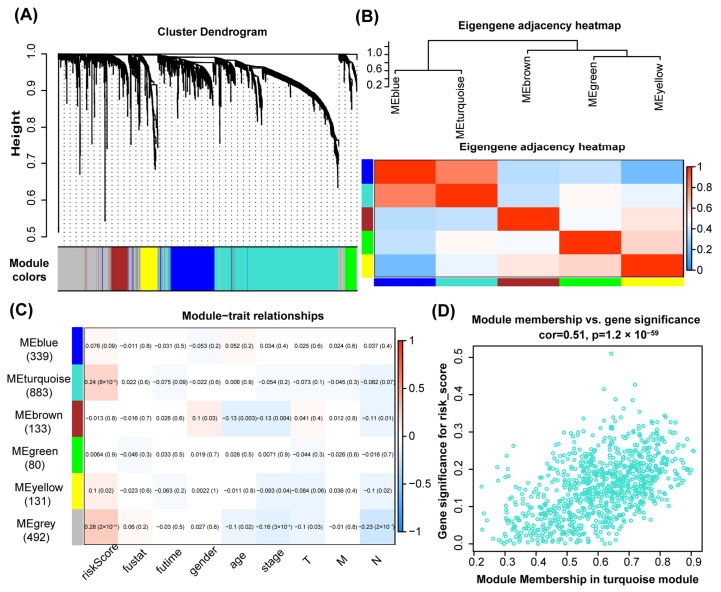
Identification of co-expression modules in COAD. (**A**) Dendrogram of genes assigned with respective module colors. Different colors represent different gene modules. (**B**) Heatmap visualizing the gene network. (**C**) Correlation between characteristic genes of distinct modules and clinicopathological variables. (**D**) Correlation analysis of the turquoise module and risk score.

Using the Topological Overlap Matrix (TOM) and a dynamic tree cut algorithm, the genes were clustered into six distinct modules (Figure 1A). The grey module consisted of genes that did not cluster with others. Within the topological overlap heatmap, each module was marked by a unique color indicating the correlation strength, with red for positive and blue for negative correlations (Figure 1B). Further analysis of each module’s correlation with the clinicopathological variables’ risk score, vital status (fustat), time to new tumor event post-treatment or time to death (futime), gender, age, stage, and TNM classification revealed significant associations. The turquoise module exhibited the strongest positive correlation with the risk score (R = 0.24, *p* = 8 × 10^−8^) (Figure 1C). In contrast, the brown module showed a robust positive association with gender (R = 0.1, *p* = 0.03) but negative correlations with age (R = −0.13, *p* = 0.003) and stage (R = −0.13, *p* = 0.004) (Figure 1C). The turquoise module, containing 883 genes, emerged as a critical determinant of CRC prognosis due to its significant association with the risk score (Figure 1D). This module’s strong linkage to tumorigenesis underscores its importance in CRC clinical outcomes.

### 3.2. Construction of the Prognostic Model Based on the Hub Genes Using LASSO and COX Regression

**Figure 2:**
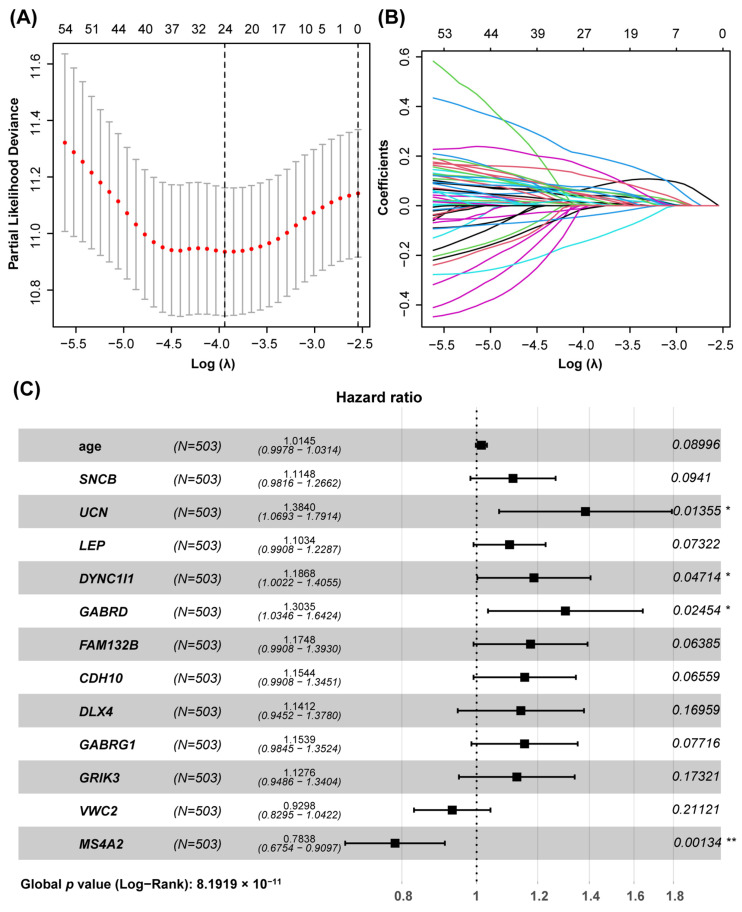
Prognostic model of genes in turquoise module. (**A**) The partial likelihood deviance for the LASSO Cox regression analysis. (**B**) LASSO coefficient plots for each independent variable, calculated using 79 genes. (**C**) Multivariable Cox analysis facilitated the screening of 12 genes for the construction of the prognostic model. * *p* < 0.05; ** *p* < 0.01.

To explore the relationship between the turquoise module and the prognosis of colon cancer patients, we constructed a prognostic signature model utilizing 883 genes from this module. Initially, univariate Cox regression analysis identified 77 genes significantly associated with patient prognosis (*p* < 0.05). Lasso regression analysis further refined this list, selecting 24 genes for inclusion while excluding 53 due to strong correlations when the cross-validation error was minimized (lambda = 0.0213) (Figure 2A,B). Multivariate Cox regression analysis subsequently identified 12 genes—10 high risk and 2 low risk—as independent prognostic factors alongside patient age (Figure 2C). A 12-gene prognostic model was formulated based on the risk coefficients of each gene, represented by the following equation: Risk score = 0.098 * Expression *_SNCB_* + 0.347 * Expression *_UCN_* + 0.103 * Expression *_LEP_* + 0.141 * Expression *_DYNC1I1_* + 0.236 * Expression *_GABRD_* + 0.168 * Expression *_FAM132B_* +0.162 * Expression *_CDH10_* + 0.138 * Expression *_DLX4_* + 0.146 * Expression *_GABRG1_* + 0.145 * Expression *_GRIK3_* + (−0.087) * Expression *_VWC2_* + (−0.257) * Expression *_MS4A2_*.

**Figure 3:**
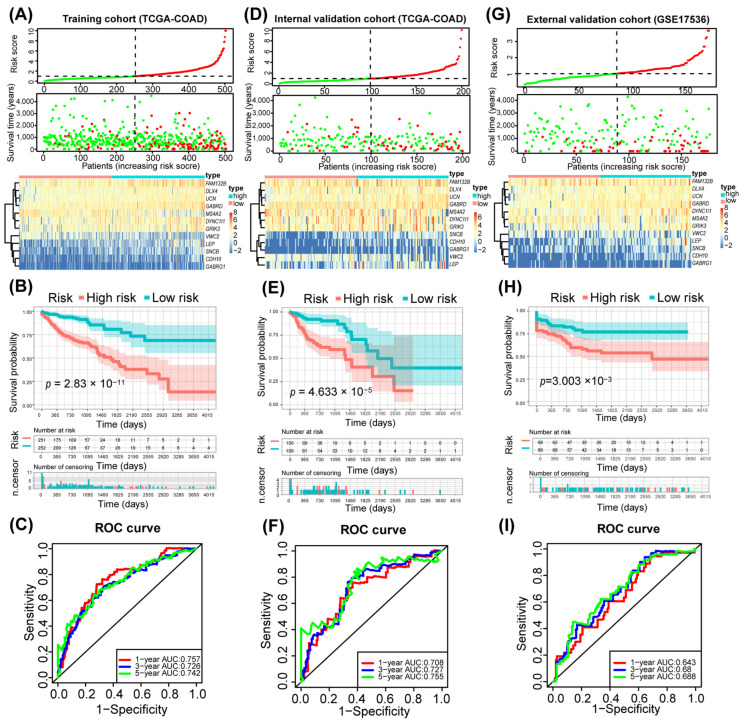
Evaluation and validation of predictive efficacy of prognostic model. (**A**,**D**,**G**) Risk score curves, survival status diagrams, and mRNA expression heatmaps demonstrate a positive correlation between the model risk score of TCGA-COAD training cohort (**A**), TCGA-COAD internal validation cohort (**D**) and GSE17536 external validation cohort (**G**) with the risk value of CRC patients. (**B**,**E**,**H**) Kaplan-Meier curves of 12-gene model for the training cohort (**B**), internal validation cohort (**E**) and external validation cohort (**H**). (**C**,**F**,**I**) ROC curves depicting the efficiency of the prognostic models in predicting 1-, 3-, and 5-year overall survival of CRC patients in the training cohort (**C**), internal validation cohort (**F**) and external validation cohort (**I**).

The risk scores were computed for each patient in the TCGA-COAD training cohort using this model. A visual representation of risk scores showed that 10 genes were overexpressed in the high-risk group and 2 in the low-risk group (Figure 3A). Patients with high-risk scores (*n* = 251) had a significantly increased mortality risk compared to those with low-risk scores (*n* = 252) (Figure 3A). Survival analysis confirmed a poorer prognosis for patients in the high-risk category (Figure 3B). The prognostic model’s accuracy was high (AUC > 0.6) for predicting the 1-, 3-, and 5-year outcomes in the TCGA-COAD cohort, as evidenced by the ROC curve (Figure 3C). Testing within the internal validation cohort TCGA-COAD and external validation cohort GSE17536 revealed that the high-risk groups had markedly elevated mortality risks and shorter overall survival compared to the low-risk groups (Figure 3D,E,G,H). The two models maintained high accuracy (AUC > 0.6) for the 1-, 3-, and 5-year prognoses in this cohort as well (Figure 3F,I). These results highlight the efficacy of the developed prognostic model in precisely predicting survival and prognosis for patients with CRC.

### 3.3. Nomogram Prognostic Model Based on Hub Genes

**Figure 4:**
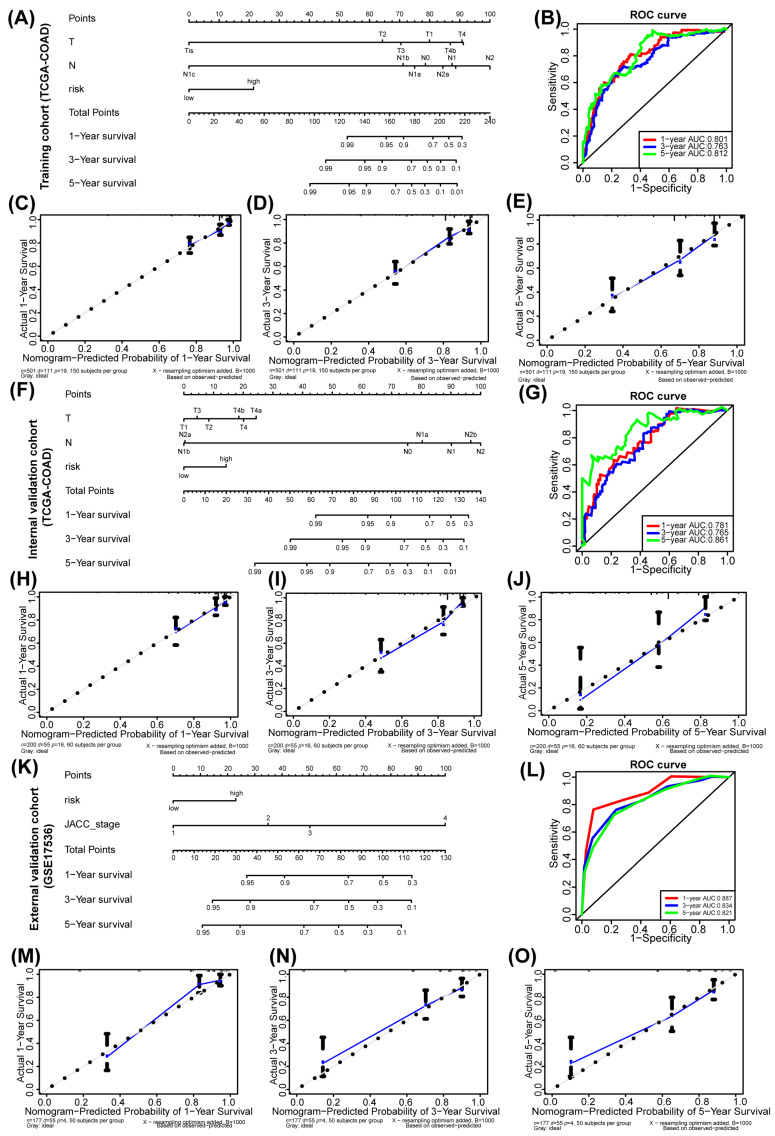
Nomogram analysis of key genes in COX prognostic model. (**A**,**F**,**K**) The nomograms for predicting 1-year, 3-year and 5-year overall survival of colorectal cancer patients in the TCGA-COAD training cohort (**A**), TCGA-COAD internal validation cohort (**F**) and GSE17536 external validation cohort (**K**). (**B**,**G**,**L**) The ROC curves for assessing the efficiency of the prognostic models in predicting 1-, 3-, and 5-year overall survival of CRC patients in the training cohort (**B**), internal validation cohort (**G**) and external validation cohort (**L**). (**C**–**E**,**H**–**J**,**M**–**O**) Calibration curves for assessing the accuracy of the nomogram in the training cohort (**C**–**E**), internal validation cohort (**H**–**J**) and external validation cohort (**M**–**O**).

A nomogram model was developed to predict the likelihood of colorectal cancer (CRC) occurrence over various survival intervals, utilizing key prognostic factors including risk status, T stage, and N stage from the TCGA-COAD training cohort (Figure 4A). This model demonstrated strong discriminative efficacy, achieving Area Under the Curve (AUC) values of 0.801, 0.763, and 0.812 for 1-, 3-, and 5-year survival, respectively (Figure 4B). The model’s predictive accuracy was further confirmed by a C-index of 0.7932, indicating high reliability. The calibration curves for 1-, 3-, and 5-year survival closely followed the 45-degree line, showcasing the model’s exceptional calibration capabilities (Figure 4C–E). This adherence underscores the nomogram’s ability to produce reliable predictions. When tested within the internal validation cohort of TCGA-COAD, the nomogram maintained its impressive discriminatory capacity (Figure 4F). The AUCs for 1-, 3-, and 5-year survival were 0.781, 0.765, and 0.861, respectively (Figure 4G), while the C-index was 0.7543. Furthermore, the calibration curves in this cohort also closely mirrored the 45-degree line (Figure 4H–J). Importantly, the nomogram also maintained its impressive discriminatory capacity within the GSE17536 external validation cohort (Figure 4K). The AUCs for 1-, 3-, and 5-year survival were 0.887, 0.834, and 0.821, respectively (Figure 4G), while the C-index was 0.8410, reinforcing its accuracy. The calibration curves in this cohort also closely mirrored the 45-degree line (Figure 4M–O), affirming the nomogram’s robust calibration performance. These results collectively highlight the nomogram model’s effectiveness in accurately predicting the probability of CRC occurrence across different survival periods. Its strong discriminative and calibration qualities provide valuable support for clinical decision-making.

### 3.4. Immune Infiltration Analysis Combining scRNA-Seq for the Two Key Genes in Prognostic Models

**Figure 5:**
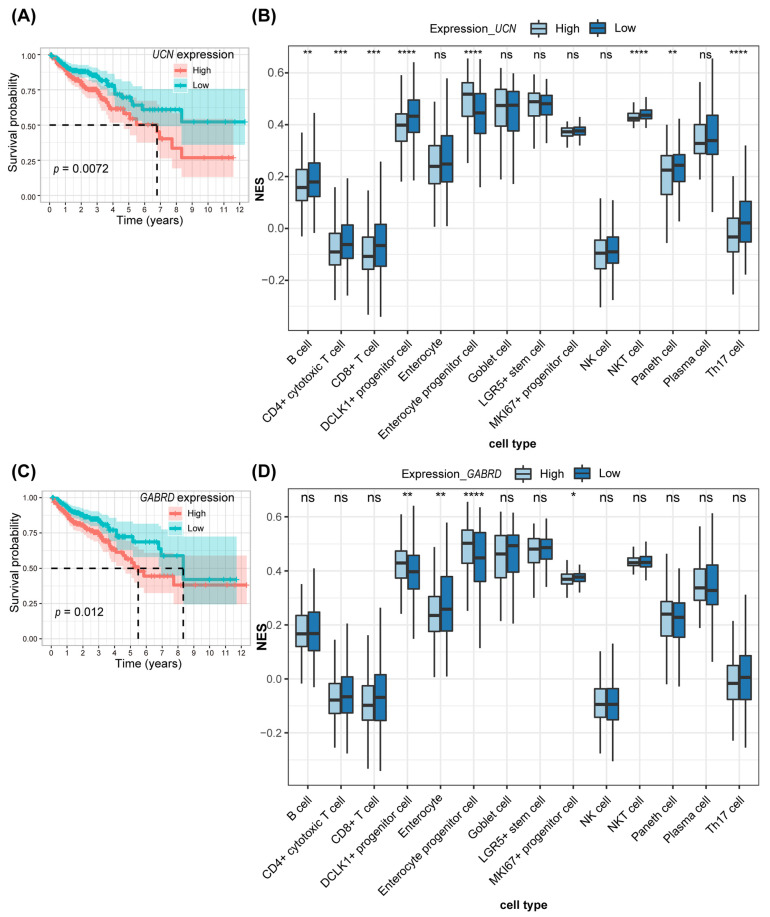
Two key genes of the prognostic models. (**A,C**) Survival analysis of *UCN* (**A**) and *GABRD* (**C**) in the TCGA-COAD database. (**B**,**D**) Immune infiltration analysis of various cell types identified by CIBERORT in the high- and low-expression groups of *UCN* (**B**) and *GABRD* (**D**) in the TCGA-COAD dataset. * *p* < 0.05; ** *p* < 0.01; *** *p* < 0.001; **** *p* < 0.0001; ns, not significant.

We conducted a thorough examination of the molecular mechanisms involving the 12 genes implicated in colorectal cancer (CRC) tumorigenesis, analyzing their expression in tumor versus normal tissues from CRC patients within the TCGA database. This revealed an upregulation of four genes and a downregulation of eight in tumor tissues (Figure S3A). Notably, increased transcript levels of *UCN* and *GABRD* were associated with a poorer prognosis (Figure 5A,C and Figure S3B–E), highlighting their potential pivotal roles in CRC regulation and the need for further investigation.

Given the limitations of standard canonical cell markers due to transcriptional heterogeneity, our analysis shifted towards identifying unique cell markers. We reanalyzed a pooled single-cell RNA sequencing (scRNA-seq) dataset including normal (number of cells = 14,170), adenoma (number of cells = 15,465), and carcinoma (number of cells = 14,530) human colon tissues. This involved filtering cells based on feature counts, performing PCA for dimensionality reduction, non-linear dimensionality reduction, and identifying cluster biomarkers to annotate cells (Figures S4 and S5). This process identified 25 clusters, distinguishing 14 cell types marked by their top 10 genes, which served as new cell markers (Figure S6). Using these markers, we quantified the abundance of the 14 identified cell types in CRC tumor tissues from the TCGA database using the CIBERSORT method. Our analysis revealed that immune cells with diminished *UCN* expression—including B cells, CD4+ cytotoxic T cells, CD8+ T cells, NKT cells, and Th17 cells—were more prevalent compared to those with high *UCN* expression (Figure 5B). Although a similar pattern was observed for GABRD, it did not reach statistical significance (Figure 5D). These findings suggest that *UCN* and *GABRD* may exert immunosuppressive effects within the CRC microenvironment, potentially mediated by their impact on the composition of immune cells. This interaction underscores the importance of their expression levels in shaping the immune landscape of CRC tumors, supporting their investigation as targets for therapeutic intervention.

### 3.5. Tumor-Promoting Action of UCN

**Figure 6:**
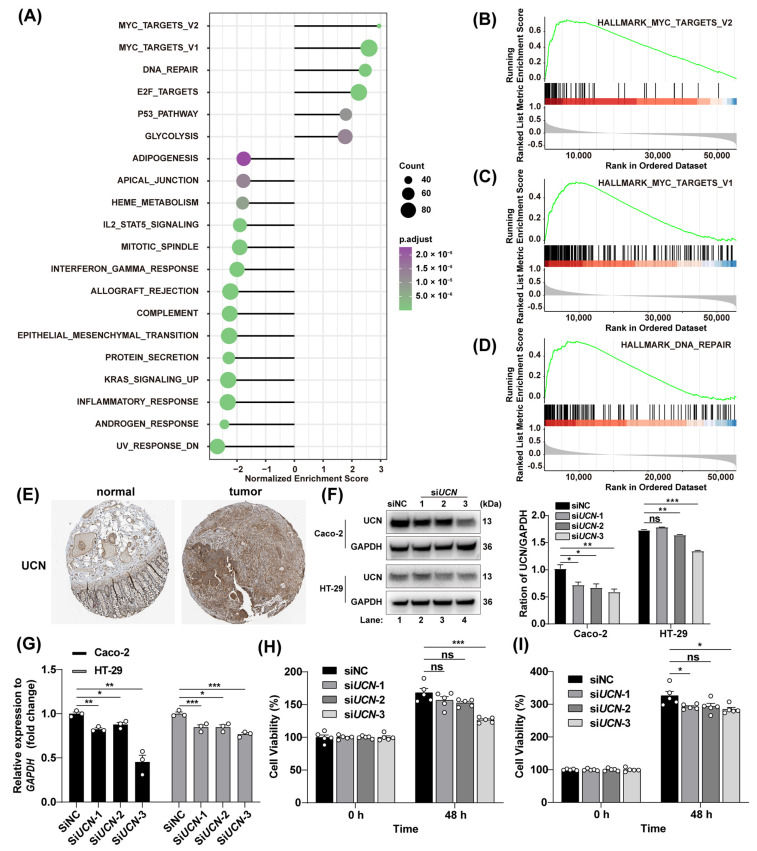
Tumor-promoting mechanism of *UCN* and tumor-inhibiting after *UCN* interference. (**A**) Gene set enrichment analysis of correlations between *UCN* and all other genes. Dot plot illustrates the overall GSEA result of *UCN* correlation. (**B**–**D**) GSEA reveals a positive association between high *UCN* expression and three key signaling pathways: MYC targets V2, MYC targets V1, and DNA repair. (**E**) Immunohistochemistry of *UCN* in colorectal cancer and normal tissue samples from the HPA database. (**F**) Immunoblot detection of UCN protein level in Caco-2 and HT-29 cells following *UCN* knockdown. (**G**) qRT-PCR demonstrates *UCN* expression levels in Caco-2 and HT-29 cells following *UCN* knockdown. (**H**,**I**) Cell viability analysis of Caco-2 and HT-29 cells post *UCN* knockdown. Data shown are mean ± SEM, and dots represent individual well of 96-well plate. Statistical analysis was performed using one-way analysis of variance (ANOVA) followed by Tukey’s multiple-comparison test. * *p* < 0.05; ** *p* < 0.01; *** *p* < 0.001; ns, not significant.

Extensive research has established the significant role of immunosuppressive factors in promoting tumorigenesis across various pathological contexts, often correlating with poor clinical outcomes in cancer patients [16,17]. Given the known immunosuppressive properties of *UCN* and *GABRD*, we hypothesized that these genes might also possess intrinsic tumorigenic capabilities. To explore this, we conducted correlation analyses to examine the interactions between *UCN* (and *GABRD* separately) and other genes. Using Gene Set Enrichment Analysis (GSEA), we identified strong correlations between *UCN* or *GABRD* and key pro-neoplastic pathways, including MYC targets V1, MYC targets V2, and DNA repair pathways, all known for their hallmark pro-tumorigenic traits (Figure 6A–D and Figure S7A–D). Further investigation revealed increased protein expression levels of *UCN* in tumor tissues compared to normal tissues in CRC patients (Figure 6E) but not *GABRD* (Figure S7E). Therefore, *UCN* is evidently the core gene of this prognostic model. Perturbation experiments targeting *UCN* functions in colonic tumor cell lines, Caco-2 and HT-29, significantly inhibited cell proliferation, providing concrete evidence of the tumorigenic mechanisms mediated by *UCN* (Figure 6F–I and Figure S7F–H). These findings illustrate that the central hub gene *UCN* within our prognostic model is not merely immunosuppressive but actively contributes to tumorigenesis. This complex characterization enhances our understanding of their roles within the CRC landscape and underscores their potential as therapeutic targets. This multifaceted approach opens new avenues for targeted interventions in colorectal cancer therapy, potentially improving patient management and outcomes.

## 4. Discussion

This study introduces a novel perspective on the complex landscape of colorectal cancer (CRC), a significant global health concern. By employing a comprehensive array of advanced analytical methodologies, our research has developed a prognostic model based on the transcriptional profiles of twelve genes. This innovative effort was facilitated by the integration of techniques such as Weighted Gene Co-expression Network Analysis (WGCNA) and LASSO Cox regression. Furthermore, the study presents a nomogram model designed to predict the likelihood of CRC occurrence over various survival durations. Constructed on the foundation of identified prognostic factors, this nomogram demonstrates remarkable accuracy and calibration, thereby enhancing the reliability of our prognostic model. This integration of sophisticated analytic methods provides a robust tool for understanding CRC dynamics, potentially guiding more effective clinical decision-making and treatment strategies.

WGCNA serves as a powerful tool in this study, enabling the detailed examination of the relationships between gene expression and disease progression by organizing genes into modules based on their shared expression patterns [12,18,19,20]. In this study, the ‘turquoise’ module has been identified as a key player in disease progression, with a strong association with risk scores. Initially, seventy-seven genes within this module correlated with the prognosis of CRC patients. Through careful application of LASSO regression and multivariate Cox regression analyses, twelve genes were selected to form the basis of a prognostic model with significant predictive power regarding survival outcomes in CRC, which was used for calculating risk scores in the TCGA-COAD training cohort, internal validation cohort, and GSE17536 external validation cohort, highlighting its clinical relevance between the high-risk groups and elevated mortality risks. These findings verified that a 12-gene model can predict the survival of CRC patients.

The tumor immune microenvironment has a profound impact on the occurrence and development of tumors. Among the 12 genes in the prognostic model, *UCN* and *GABRD* emerged as critical due to their association with survival rates and their roles within the complex immune environment of the tumor [21,22,23]. The integration of single-cell RNA sequencing (scRNA-seq) data significantly enhances the understanding of the tumor immune microenvironment of this study. Unlike bulk RNA-seq, scRNA-seq provides a detailed view of gene expression at the individual cell level [24,25], which is instrumental in identifying unique cell populations and uncovering novel cellular types and states. In this research, scRNA-seq was pivotal in defining distinctive immune cell populations specific to colorectal cancer (CRC), thereby refining the accuracy of immune infiltration analyses. Upon immune infiltration analyses, immune cells with decreased *UCN* and *GABRD* expression were more prevalent compared to those with high expression; *UCN* and *GABRD* perform immunosuppressive effects within the CRC microenvironment. Their impact on the prevalence of specific immune cell types suggests the role of immunosuppressive factors such as *UCN* and *GABRD* in tumor-promoting properties, presenting valuable targets for therapeutic intervention. Additionally, the study sheds light on the tumorigenic capabilities of *UCN* and *GABRD*, linking these genes to pivotal pathways known to drive cancer progression, such as MYC targets V1, MYC targets V2, and DNA repair pathways. Considering the increased protein expression levels of *UCN* in tumor tissues compared to normal tissues in the CRC patients, *UCN* serves as the key gene in the prognostic model. Perturbation of *UCN* expression notably curtailed tumor cell proliferation, underscoring their potential as a target for innovative cancer therapies.

## 5. Conclusions

This study constructed a 12-gene prognostic model using WGCNA and used LASSO regression analyses to predict the CRC patient outcomes. Combined with immune infiltration analysis and gene perturbation assays, *UCN* is the key gene promoting tumor development in the model. In conclusion, this model not only supports the early diagnosis and prognosis of CRC patients but also identified a key target for treatment by exploring the core genes involved in tumor development and the tumor immune microenvironment.

## Data Availability

The bulk RNA-seq dataset TCGA-COAD was obtained from The Cancer Genome Atlas (https://portal.gdc.cancer.gov/, accessed on 1 January 2023). The bulk RNA-seq dataset GSE17536 was accessed from the GEO database (https://www.ncbi.nlm.nih.gov/geo, accessed on 15 January 2023). The CRC scRNA-seq dataset GSE161277 was also accessed from the GEO database (https://www.ncbi.nlm.nih.gov/geo, accessed on 15 January 2023). The data generated by this study are available from the Supplementary Materials. The R software packages utilized in this study are freely and open source. Source code for bulk RNA-seq and scRNA-seq are available at GitHub (https://github.com/liujialeo/Prognostic_model_of_COAD, accessed on 8 August 2023).

## Supplementary Materials

The following supporting information can be downloaded at: https://www.mdpi.com/article/10.3390/genes15091139/s1, Figure S1: Analysis of differentially expressed mRNA in TCGA-COAD dataset; Figure S2: Determination of the soft-thresholding power in the WGCNA; Figure S3: Gene expression and survival analysis of the 12 prognostic genes in the TCGA-COAD dataset; Figure S4: Quality control for single-cell data filtering; Figure S5: Expression profiles of marker genes across cell types; Figure S6: Overview of single cells from normal, adenoma and carcinoma cancer; Figure S7: Tumor-promoting mechanism of GABRD and tumor-inhibiting after GABRD interference; Table S1: Primers used in this study; Table S2: The genes used to construct the model and their corresponding risk coefficient.
