# Supplementary material for: UCN-Centric Prognostic Model for Predicting Overall Survival and Immune Response in Colorectal Cancer
Source: Genes (Basel). 2024 Aug 29;15(9):1139. doi: 10.3390/genes15091139 (PMC11430869; doi:10.3390/genes15091139)
Supplement: Supplementary file 1 [file genes-15-01139-s001.zip › genes-3145516-supplementary.pdf]

Supplementary Table S1. Primers used in this study

Gene	Primer	Sequence (5'-3')
UCN	Forward	AACCCTTCTCTGTCCATTGACCT
	Reverse	ACCGAGTCGAATATGATGCGGTTC
GAPDH	Forward	AGCCACATCGCTCAGACAC
	Reverse	ACCAGGCGCCCAATACGA

Supplementary Table S2. The genes used to construct the model and their corresponding risk coefficient.

ID	coef	HR	HR.95L	HR.95H	P-value
SNCB	0.098	1.103	0.973	1.251	0.126
UCN	0.347	1.415	1.094	1.830	0.008
LEP	0.103	1.108	0.996	1.233	0.060
DYNC1I1	0.141	1.151	0.980	1.352	0.087
GABRD	0.236	1.266	1.012	1.586	0.039
FAM132B	0.168	1.183	1.000	1.400	0.050
CDH10	0.162	1.176	1.011	1.367	0.035
DLX4	0.138	1.148	0.955	1.380	0.143
GABRG1	0.146	1.157	0.988	1.355	0.071
GRIK3	0.145	1.156	0.979	1.365	0.088
VWC2	-0.087	0.917	0.819	1.026	0.131
MS4A2	-0.257	0.774	0.667	0.897	0.001

Supplementary figure legend

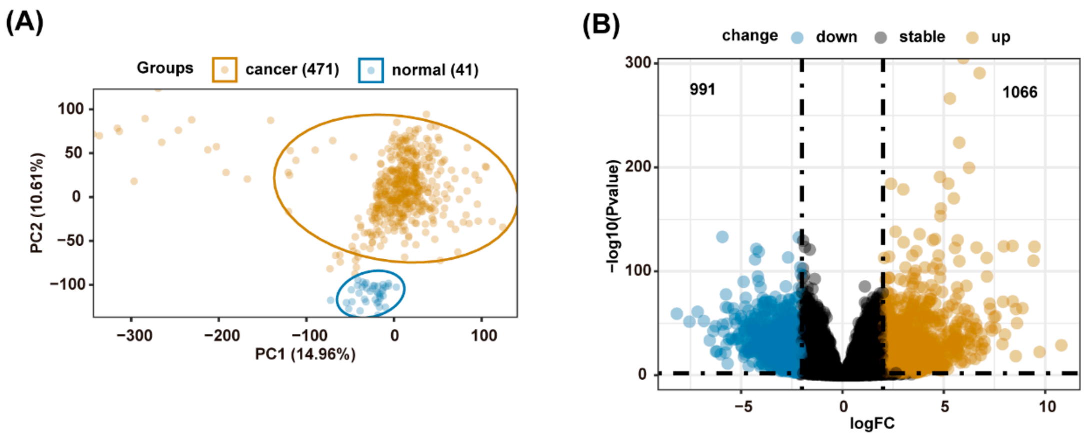

Supplementary Figure S1. Analysis of differentially expressed mRNA in TCGA-COAD dataset. (A) Principal Component Analysis (PCA) plot visualizing the distribution and variance of mRNA expression profiles. **(B)** Volcano plot illustrating the differentially expressed mRNAs, with significantly upregulated genes marked in orange and significantly downregulated genes marked in blue.

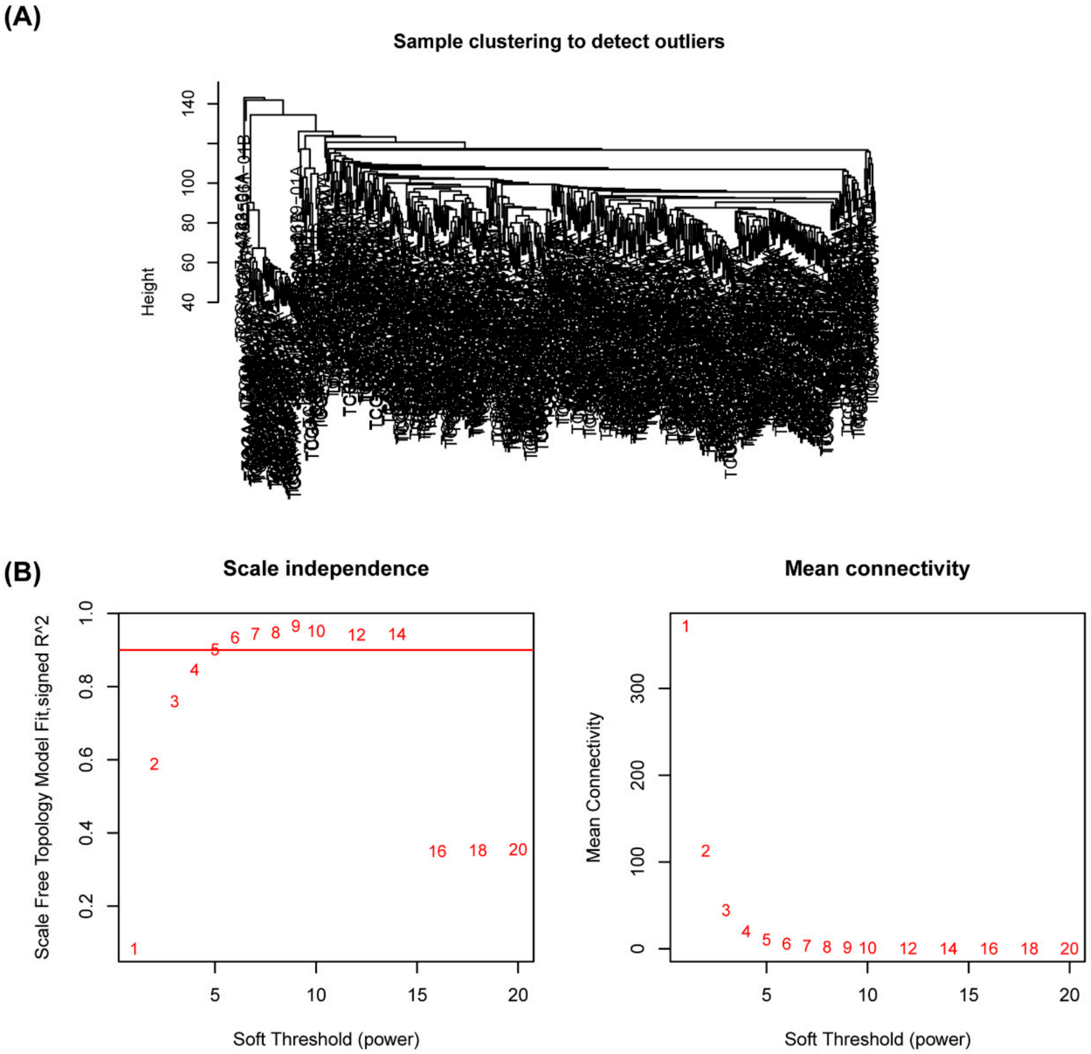

Supplementary Figure S2. Determination of the soft-thresholding power in the WGCNA. (A) Clustering dendrogram of samples, used to detect and exclude outliers. **(B)** Analysis of the scale-free fit index for various soft-thresholding powers (β). The soft-thresholding power selected is the first one for which the scale-free topology fit index exceeds the threshold of 0.9, thus ensuring a scale-free network.

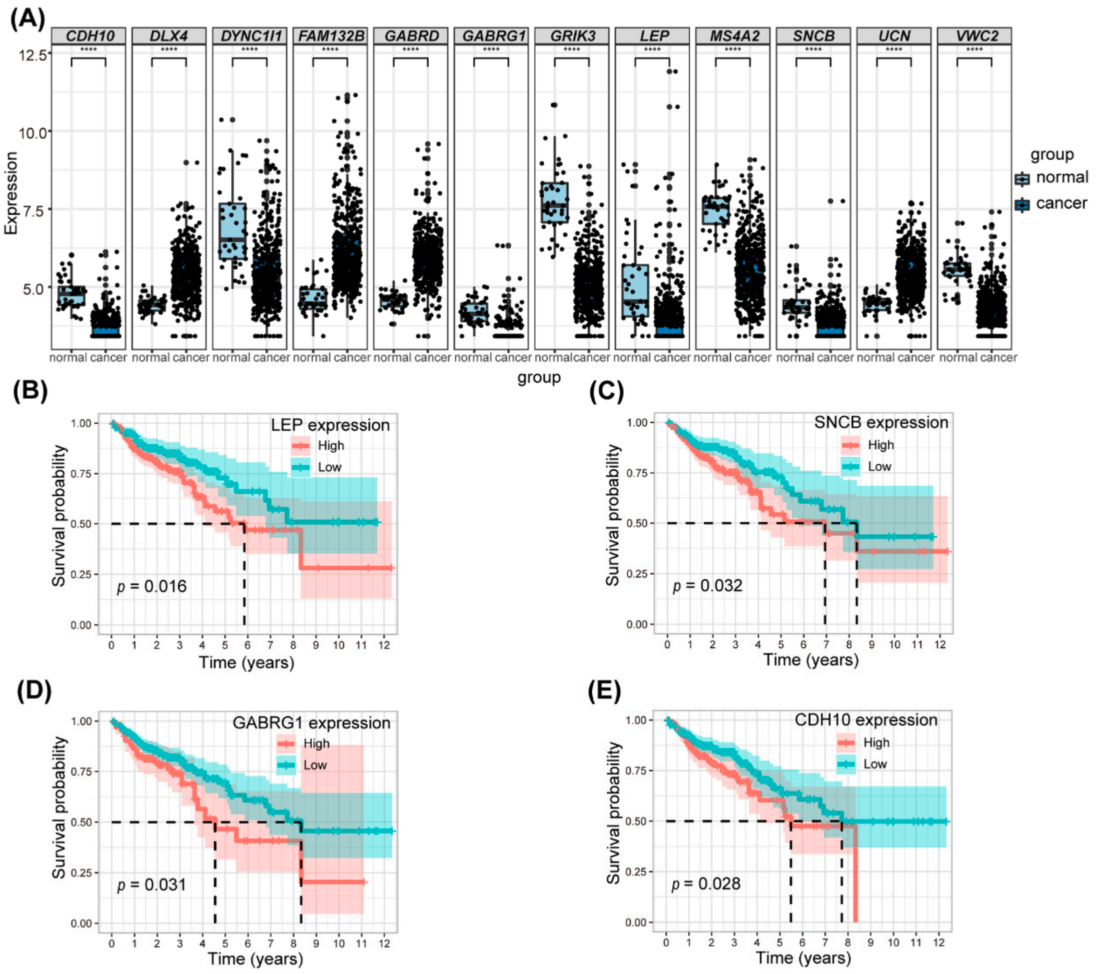

Supplementary Figure S3. Gene expression and survival analysis of the 12 prognostic genes in the TCGA-COAD dataset. (A) Boxplots representing the expression levels of the 12 genes comprising the prognostic model in the TCGA-COAD dataset. **(B-E)** Kaplan-Meier survival curves illustrating the association between the expression of four of these genes (*LEP*, *SNCB*, *GABRG1*, and *CDH10*) and overall survival in the TCGA-COAD dataset. High expression (red) and low expression (green) groups are compared. **** $p < 0.0001$.

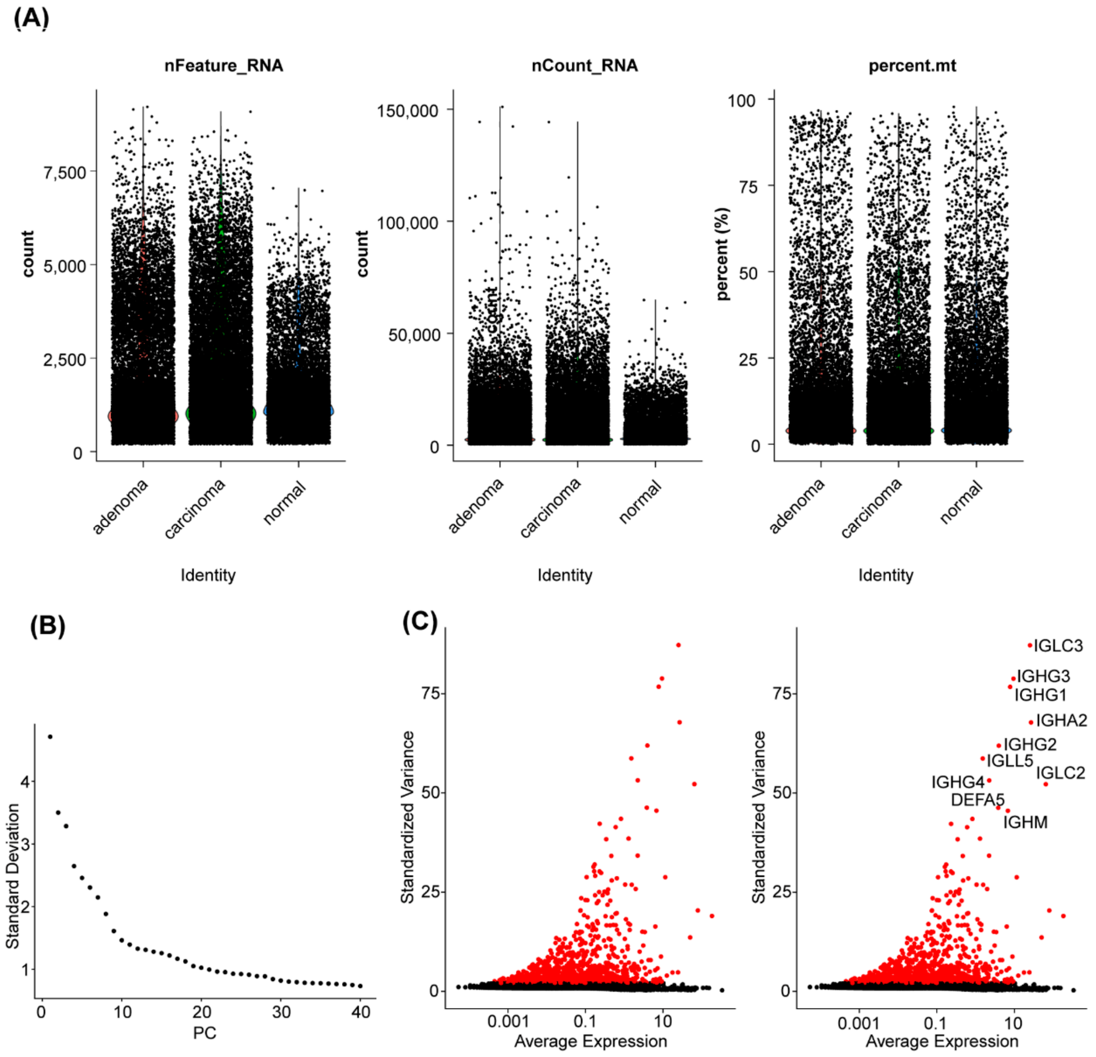

Supplementary Figure S4. Quality control for single-cell data filtering. (A) Violin plots demonstrating the distribution of quality control metrics across cells, including the number of detected genes, the total counts per cell, and the proportion of counts mapping to mitochondrial genes. **(B)** Principal Component Analysis (PCA) illustrating the distribution of cells in reduced-dimensional space, allowing for the visualization of cell clustering based on gene expression profiles. **(C)** Identification of highly variable features.

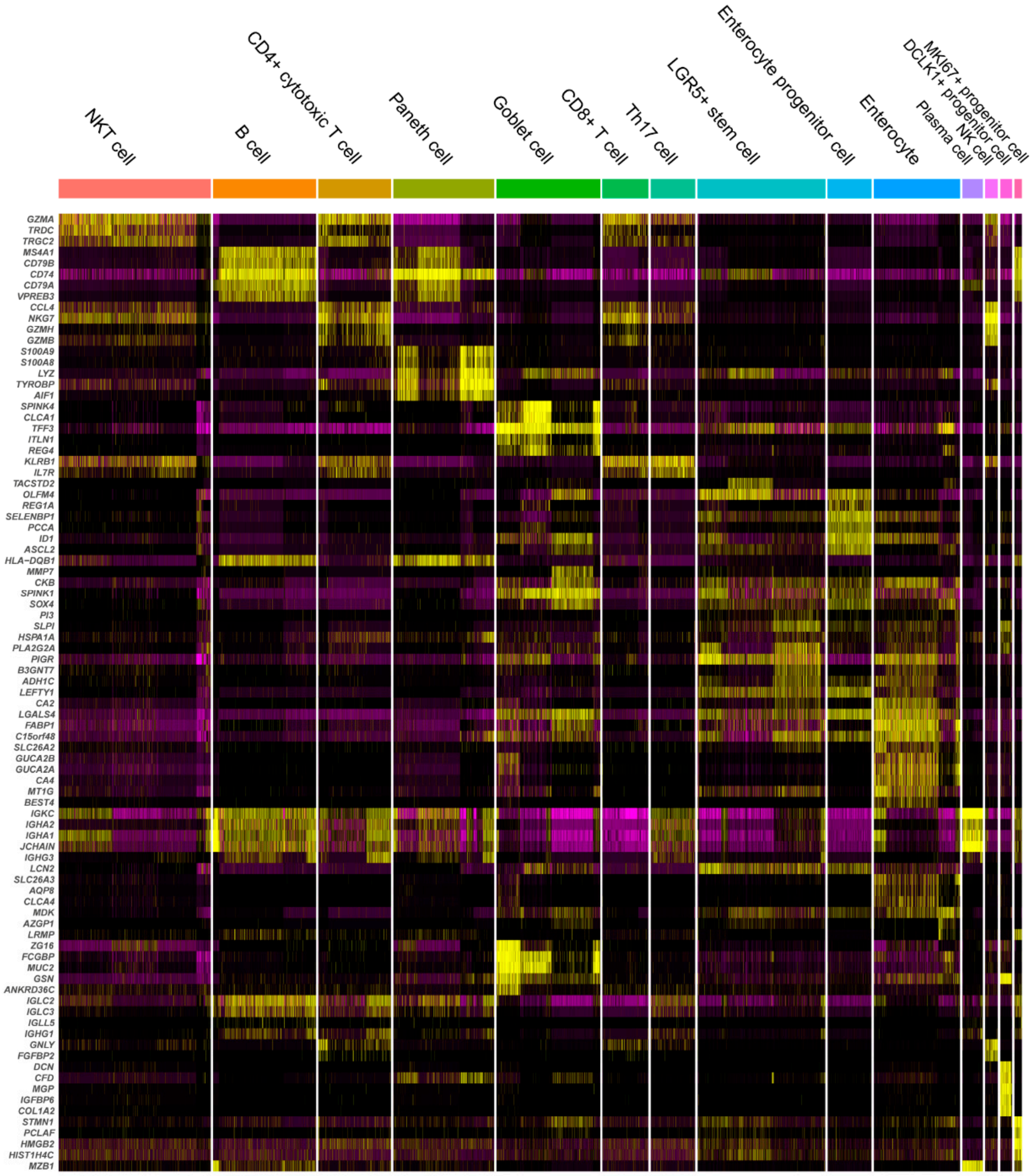

Supplementary Figure S5. Expression profiles of marker genes across cell types. Heatmap representation of the expression levels of the top five marker genes for each of the 14 identified cell types in the single-cell RNA sequencing dataset. Each column represents a cell type, and each row corresponds to a gene. The color intensity in the heatmap indicates the gene expression level, with yellow indicating high expression and purple indicating low expression. This visualization aids in characterizing cell type-specific gene expression patterns and validates the cell type identification.

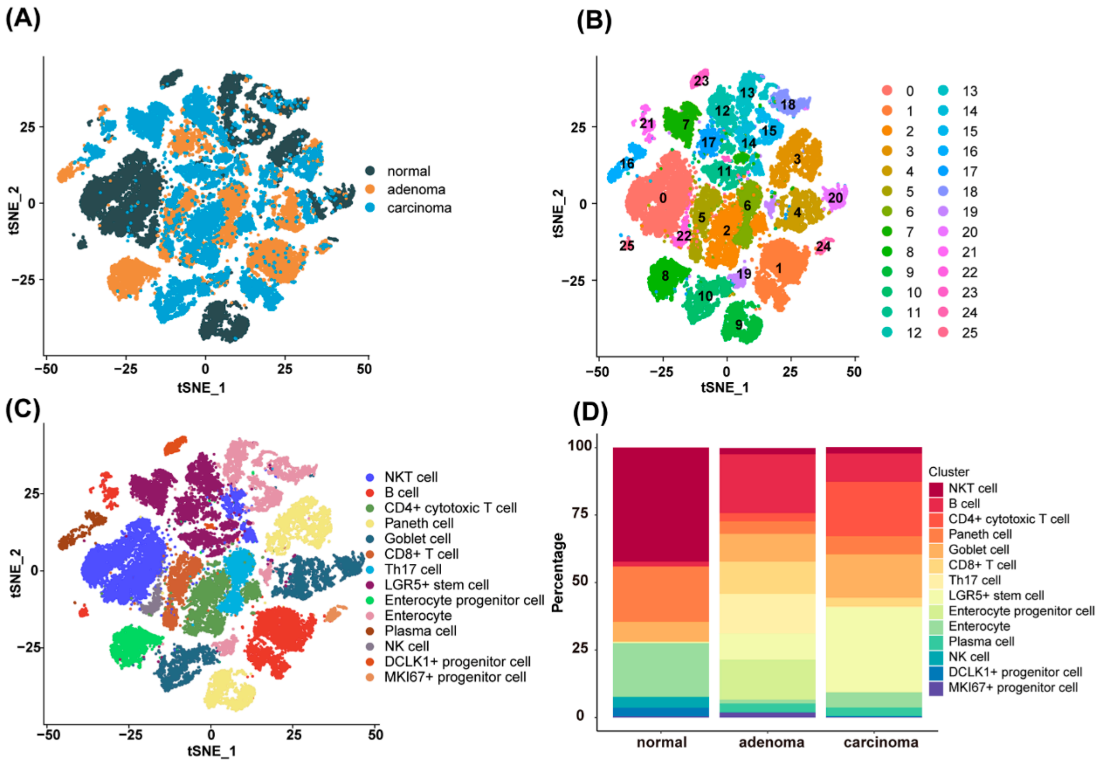

Supplementary Figure S6. Overview of single cells from normal, adenoma and carcinoma cancer. (A) t-SNE plot illustrating single-cell RNA sequencing data obtained from the publicly available GSE161277 dataset, representing normal, adenoma, and carcinoma colon tissues. Each dot corresponds to a single cell, with different colors indicating the tissue types. **(B)** t-SNE plot displaying a total of 37,640 cells divided into 26 distinct clusters. Each color represents a different cluster. **(C)** Identification of 14 cell types using specific marker genes, as displayed on the t-SNE plot. Each cell type is represented by a different color. **(D)** Stacked bar plot demonstrating the relative percentages of the 14 identified cell types across the different tissue types. Each color in the bar represents a specific cell type, and the height of the colored segment corresponds to the percentage of cells of that type.

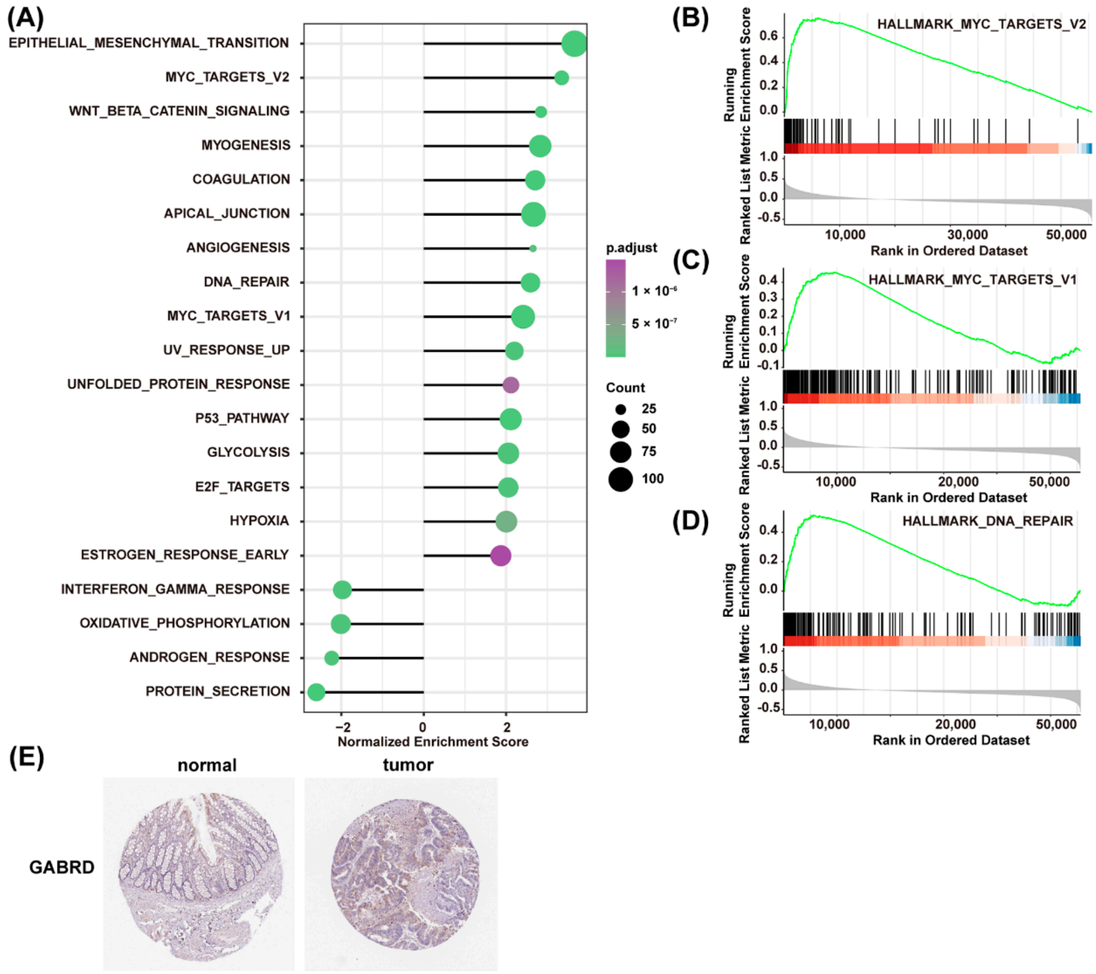

Supplementary Figure S7. Tumor-promoting mechanism of *GABRD* and tumor-inhibiting after *GABRD* interference.

(A) Gene set enrichment analysis of correlations between *GABRD* and all other genes. Dot plot illustrates the overall GSEA result of *GABRD* correlation. **(B-D)** GSEA reveals a positive association between high *GABRD* expression and three key signaling pathways: MYC targets V2, MYC targets V1, and DNA repair. **(E)** Immunohistochemistry of *GABRD* in colorectal cancer and normal tissue samples from the HPA database.
